# Changes in circulating microRNA and arterial stiffness following high‐intensity interval and moderate intensity continuous exercise

**DOI:** 10.14814/phy2.14431

**Published:** 2020-05-01

**Authors:** Ryan M. Sapp, Catalina A. Chesney, Lauren E. Eagan, William S. Evans, Evelyn M. Zietowski, Steven J. Prior, James M. Hagberg, Sushant M. Ranadive

**Affiliations:** ^1^ Department of Kinesiology School of Public Health University of Maryland College Park MD USA; ^2^ Department of Biology University of Maryland College Park MD USA; ^3^ Baltimore Veterans Affairs Geriatric Research Education and Clinical Center Baltimore MD USA

**Keywords:** Arterial stiffness, Augmentation index, High‐intensity interval exercise, MicroRNA, Pulse wave velocity

## Abstract

High‐intensity interval (HII) exercise elicits distinct vascular responses compared to a matched dose of moderate intensity continuous (MOD) exercise. However, the acute effects of HII compared to MOD exercise on arterial stiffness are incompletely understood. Circulating microRNAs (ci‐miRs) may contribute to the vascular effects of exercise. We sought to determine exercise intensity‐dependent changes in ci‐miR potentially underlying changes in arterial stiffness. Ten young, healthy men underwent well‐matched, 30‐min HII and MOD exercise bouts. RT‐qPCR was used to determine the levels of seven vascular‐related ci‐miRs in serum obtained immediately before and after exercise. Arterial stiffness measures including carotid to femoral pulse wave velocity (cf‐PWV), carotid arterial compliance and β‐stiffness, and augmentation index (AIx and AIx75) were taken before, 10min after and 60min after exercise. Ci‐miR‐21‐5p, 126‐3p, 126‐5p, 150‐5p, 155‐5p, and 181b‐5p increased after HII exercise (*p* < .05), while ci‐miR‐150‐5p and 221‐3p increased after MOD exercise (*p* = .03 and 0.056). One hour after HII exercise, cf‐PWV trended toward being lower compared to baseline (*p* = .056) and was significantly lower compared to 60min after MOD exercise (*p* = .04). Carotid arterial compliance was increased 60min after HII exercise (*p* = .049) and was greater than 60min after MOD exercise (*p* = .02). AIx75 increased 10 min after both HII and MOD exercise (*p* < .05). There were significant correlations between some of the exercise‐induced changes in individual ci‐miRs and changes in cf‐PWV and AIx/AIx75. These results support the hypotheses that arterial stiffness and ci‐miRs are altered in an exercise intensity‐dependent manner, and ci‐miRs may contribute to changes in arterial stiffness.

## Graphical abstract

We identified a reduction in central artery stiffness and an increase in carotid arterial compliance one hour after acute high intensity interval (HII) cycling exercise, that was not seen after moderate intensity continuous cycling (MOD). HII and MOD elicited distinct increases in the concentrations of circulating microRNAs (ci‐miRs) with potential regulatory roles in arterial stiffness. Significant correlations between some of the changes in ci‐miRs and measures of arterial stiffness suggest potential mechanistic roles for ci‐miRs.

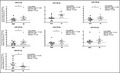

## 1 INTRODUCTION

Progressively increased stiffening of the large arteries occurs with aging and contributes to the development of cardiovascular disease (CVD) (Shirwany & Zou, 2010). The minimization of arterial stiffening is a major target for the prevention of CVDs, though the cellular and molecular mechanisms underlying changes in arterial stiffness are not completely understood. Exercise can elicit acute alterations in arterial stiffness (Mutter, Cooke, Saleh, Gomez, & Daskalopoulou, 2017; Pierce, Doma, & Leicht, 2018), providing a useful model to study potential underlying mechanisms. Both long term changes with aging and acute exercise‐induced changes in arterial stiffness may result from a combination of altered hemodynamics, sympathetic stimulation, inflammatory factors, reactive oxygen species (ROS), vascular tone, and other circulating factors (Atkinson, Carter, et al., 2015; Atkinson, Lewis, et al., 2015; Dawson, Green, & Cable, 2013,; Heffernan, Edwards, Rossow, Jae, & Fernhall, 2007; Johnson & Wallace, 2012; Mutter et al., 2017; Seals, Nagy, & Moreau, 2019; Shirwany & Zou, 2010). High‐intensity endurance exercise results in greater and unique changes in many of these stimuli, which are proposed to explain the larger impairment in endothelial function often observed immediately after higher intensity compared to low‐moderate intensity exercise (Antunes et al., 2019; Birk et al., 2013; Dawson et al., 2013,; Johnson, Mather, Newcomer, Mickleborough, & Wallace, 2012; McClean, Harris, Brown, Brown, & Davison, 2015; Peake et al., 2005). It is less clear whether there are exercise intensity‐dependent changes in arterial stiffness and what factors may potentially contribute to such differences (Mutter et al., 2017; Pierce et al., 2018).

MicroRNAs (miRs) are post‐transcriptional regulators of gene expression that have been implicated as a novel class of molecules important in maintaining arterial health and mediating cardiovascular responses to exercise (Bartel, (2018); Donaldson, Lao, & Zeng, 2018; Fernández‐Hernando & Suárez, 2018; Lee & Chiu, 2019; Nanoudis, Pikilidou, Yavropoulou, & Zebekakis, 2017; Sapp, Shill, Roth, & Hagberg, 2017; Sapp & Hagberg, 2019). Circulating miRs (ci‐miRs) released from cells into the bloodstream are proposed as novel biomarkers and mediators of cardiovascular exercise responses, as well as CVD (Johnson, 2019; Njock & Fish, 2017; Sapp & Hagberg, 2019; Sapp et al., 2017,). The levels of specific ci‐miRs correlate with arterial stiffness in humans (Deng et al., 2017; Parthenakis et al., 2017; Van Craenenbroeck et al., 2016). Additionally, in vitro and animal experiments have shown that changes in specific vascular stimuli such as shear stress and inflammation affect the expression and release of microRNAs from vascular cells, suggesting these molecules may play roles in exercise intensity‐dependent changes in arterial stiffness (Alexy, Rooney, Weber, Gray, & Searles, 2014; Donaldson et al., 2018; Hale et al., 1843; Lee & Chiu, 2019; Schmitz et al., 2019; Zhou et al., 2013).

Therefore, we sought to determine the effects of a moderate intensity continuous and a high‐intensity interval (HII) exercise bout on the concentrations of several ci‐miRs with potential roles in the regulation of arterial stiffness. The responses of central and large arterial stiffness were simultaneously determined in order to explore associations with the changes in ci‐miRs. We hypothesized that HII exercise would elicit greater changes in ci‐miRs and arterial stiffness compared with a matched bout of moderate intensity continuous exercise, and that the responses of these variables would be significantly correlated.

## 2 METHODS

### 2.1 Ethical approval

Prior to participant enrollment, all testing procedures were approved by the University of Maryland Institutional Review Board (IRB).

### 2.2 Participants

Apparently healthy men between the ages of 18 and 39 who were moderately active (reporting physical activity where heart rate (HR) was continuously elevated for at least 30 min on 1–4 days per week) were recruited. On average, participants reported exercising on 3.5 ± 0.8 days/week for 1.3 ± 0.7 hr/session. Participants reported being African American (*n* = 5) or Caucasian (*n* = 5). The study consisted of three morning visits to our laboratory. Prior to each visit, subjects were required to refrain from food and drink other than water overnight (≥10 hr), as well as NSAIDs, alcohol, and exercise for at least 24 hr. During the first visit, participants filled out paperwork including the IRB‐approved informed consent, and health and physical activity questionnaires. Following demographic testing, a resting blood draw was taken from an antecubital vein for blood chemistry analysis. Participants were excluded if they had any one of the following: body mass index (BMI) >30 kg/m^2^, brachial systolic blood pressure (BP) >140 mmHg, diastolic BP > 90 mmHg, use of antihypertensive medication, or presence of cardiovascular disease. Information on participants and procedures have been previously reported (Sapp et al., 2019).

### 2.3 Exercise visits

At the end of the first visit, participants completed a peak power output (PPO) test on a leg cycle ergometer (Monark Ergomedic 894E). The test consisted of cycling at 70–80 rpm with 2‐min stages of increasing load until volitional exhaustion. The initial load consisted of 1.6 kg for the first stage, with each subsequent stage increasing by 0.3 or 0.6 kg. An individual's PPO was determined as the load at which they were unable to maintain at least 70 rpm. Appropriate loads for the subsequent exercise bouts were then calculated based on each participant's PPO. The 30‐min exercise bouts completed during visits 2 and 3 consisted of a moderate intensity continuous (MOD) bout at 60% PPO and a high‐intensity interval (HII) bout beginning with 6 min at 40% PPO followed by 3‐min intervals at 85% PPO interspersed with 4‐min intervals at 40% PPO. Participants maintained a cadence of 80 rpm throughout. Importantly, exercise bouts were matched for average PPO and, therefore, exercise volume. A chest strap HR monitor (POLAR T31) was used to record HR during each minute of exercise.

To avoid potential carryover effects of exercise, visits were separated by at least one week. The HII and MOD bouts were performed in a randomized, counterbalanced order, such that half of the participants completed the HII bout first and half completed the MOD bout first. Upon entering the laboratory for visits 2 and 3, participants laid supine for 10 min before the baseline vascular testing and blood draw. Immediately after completion of exercise, participants returned to the phlebotomy bed and laid supine for the post‐exercise blood draw and preparation for arterial stiffness assessments.

### 2.4 Blood collection

Blood was drawn from an antecubital vein immediately before and after exercise while participants lay in the supine position. Approximately 10 ml of blood was collected into a serum separator tube and allowed to sit at room temperature for 45 min before centrifugation at 1,500g for 15 min at 4ºC. Serum was pipetted into 500 µl aliquots on ice and transferred to a −80ºC freezer for storage.

### 2.5 Circulating microRNA

Serum samples were thawed at room temperature and centrifuged at 16,000g for 10 min at 4ºC to pellet cell debris. The miRNeasy serum/plasma kit (Qiagen) was used to isolate RNA from 50 µl of serum. In a modification to the manufacturer's protocols, 20 volumes of Qiazol lysis reagent and 4 volumes of chloroform were added to the serum. An equal amount of C. elegans microRNA (C. elegans miR‐39) was spiked into each sample after the addition of Qiazol to act as a control for isolation and PCR efficiency. Total RNA was eluted using 14 µl of RNAse‐free water. The miScript II RT kit (Qiagen) was used to perform reverse transcription using 5 µl of RNA. The final reaction mixture was diluted in 200 µl of RNAse‐free water. Each RT‐qPCR reaction was performed using 2.5 µl of the resulting cDNA and the miScript SYBR Green PCR Kit (Qiagen). For each ci‐miR target, all samples were run on the same 96‐well plate in duplicate on an ABI 7300 Real‐Time PCR System (Applied Biosystems). Primers were used for the specific amplification of each ci‐miR (Qiagen; miR‐21‐5p: MS00009079, miR‐126‐3p: MS00003430, miR‐126‐5p: MS00006636, miR150‐5p: MS00003577, miR‐155‐5p: MS00031486, miR‐181b‐5p: MS00006699, miR‐221‐3p: MS00003857). The miR strands (3p versus. 5p) studied were chosen because they are reported in the literature as the dominantly expressed strand; the complimentary strand being degraded. For miR‐126, both strands are highly expressed in endothelial cells and are found circulating in abundance (Fish et al., 2008; Harris, Yamakuchi, Ferlito, Mendell, & Lowenstein, 2008; Wang et al., 2008). The 2^−ΔΔCT^ method of relative quantification was used, where for each miR in each sample, Δ cycle threshold (CT) = CT of miR—CT of spike‐in control miR; ΔΔCT = ΔCT for individual's post‐exercise sample—ΔCT of individual's respective baseline sample.

### 2.6 Pulse wave velocity

Cuff‐based applanation tonometry (SphygmoCor, AtCor Medical, Sydney, Australia) was performed to obtain pressure waveforms in the femoral artery, while the carotid pressure waveform was obtained using hand‐held tonometer. A standard tape measure was used on the right side of the body to calculate straight‐line distances from the sternal notch to the carotid and femoral artery sites and from the femoral artery pulse site to the top of the thigh BP cuff. These distances were used to determine the path length for carotid‐femoral pulse wave velocity (cf‐PWV), a measure of central stiffness. Waveforms were collected by a high‐fidelity tonometer for a ten second capture period. Cf‐PWV is calculated as Δ distance (m)/Δ time (s), where Δ time is the time delay between pulse arrivals at the carotid and femoral arteries.

### 2.7 Carotid arterial compliance and β‐stiffness

The right common carotid artery was imaged via ultrasound using a 7.5 MHz linear‐array probe (Aloka, Hitachi). Heart rate was monitored using a three‐lead ECG. Image analysis and calculations of arterial compliance and β‐stiffness were carried out using an automated wall detection echo‐tracking software system. Measurements were calibrated to brachial artery BP (measured by sphygmomanometer).

### 2.8 Augmentation index

Cuff based applanation tonometry (SphygmoCor, AtCor Medical, Sydney, Australia) was used to reconstruct an aortic pressure waveform from the brachial artery pressure waveform using a generalized validated transfer function. Augmentation index (AIx) was determined as the ratio of the difference between the first and second systolic peaks of the arterial waveform to the total pulse pressure, expressed as a percentage. Since AIx is influenced by HR variation, AIx values were also normalized to a HR of 75 beats/min (AIx75).

### 2.9 Statistical analyses

Ci‐miRs were analyzed using two‐way ANOVAs with exercise intensity and time as factors, followed by pre‐planned post‐hoc Fisher's LSD tests comparing exercise time points within and between each exercise intensity separately. Measures of arterial stiffness were analyzed using repeated measures two‐way ANOVAs and pre‐planned Fisher's LSD tests. Correlations between exercise‐induced changes in HR and vascular measures with ci‐miRs were determined by calculating Spearman correlation coefficients. Two‐way repeated measures ANOVAs were also performed on HR and BP change data at the 10 and 60 min time points after exercise as compared to supine rest before exercise (baseline). All tests were two‐sided. Statistical significance was set at *p* < .05, while 0.05 < *p*<.10 was considered borderline significant.

## 3 RESULTS

### 3.1 Participants

Ten young, healthy men were recruited and completed all testing. Detailed demographics are shown in Table 1 (also previously provided (Sapp et al., 2019)).

**Table 1:** Demographic information

Subject Characteristics (*n* = 10)	
Age, y	22 ± 2
BMI, kg/m^2^	24 ± 3
Peak Power Output, kgm/s	29 ± 5
Resting HR, bpm	66 ± 8
SBP, mmHg	127 ± 7
DBP, mmHg	69 ± 10
MAP, mmHg	88 ± 7
Glucose, mg/dl	87 ± 6
Hemoglobin A1c, %	5.0 ± 0.4
Total cholesterol, mg/dl	154 ± 26
HDL‐C, mg/dl	55 ± 12
LDL‐C, mg/dl	83 ± 15
VLDL‐C, mg/dl	14 ± 5
Triglycerides, mg/dl	68 ± 22

Means ± *SD*.

BMI, body mass index; HR, heart rate; SBP, brachial systolic blood pressure; DBP, brachial diastolic blood pressure; MAP, mean brachial arterial pressure; HDL‐C, high‐density lipoprotein cholesterol; LDL‐C, low‐density lipoprotein cholesterol; VLDL‐C, very low‐density lipoprotein cholesterol.

### 3.2 Heart rate and blood pressure

The HII and MOD exercise bouts were well‐matched, eliciting average HRs of 152 ± 7 and 155 ± 10 bpm, respectively (*p* = .54) (see reference Sapp et al., 2019). The peak HR was significantly higher during HII compared to MOD (186 ± 8 versus 170 ± 11 bpm, *p* = .0001). Post‐exercise changes in HR, brachial arterial BP, and aortic BP from baseline are reported in Table 2. We have previously reported mean arterial pressure values (Sapp et al., 2019), however, given the important relationship between arterial BP and stiffness, here we have reported changes to both systolic and diastolic pressures. Heart rate was significantly elevated 10 and 60 min after both the MOD (*p* < .0001 and *p* = .048) and HII bouts (*p* < .0001 and *p* = .0007), and to a greater degree 10 min after HII compared with MOD (*p* = .005). There were no significant changes in brachial arterial BP as compared to baseline after either exercise bout, though the change in brachial systolic BP 60 min after HII was significantly different as compared to the change 60 min after MOD (*p* = .02). There was a significant increase in aortic diastolic BP 10 min after (*p* = .009) and a significant decrease in aortic systolic BP 60 min after HII exclusively (*p* = .045). The decrease in aortic diastolic BP 10 min after HII exercise was also significantly different compared to the change 10 min after MOD (*p* = .045).

**Table 2:** Heart rate, brachial blood pressure, and aortic blood pressure changes from baseline

		10 min. post	60 min. post	Two‐way ANOVA *p*‐value		
				Time	Intensity	Interaction
Heart Rate	MOD	+17.3 ± 7.6^*^	+4.9 ± 10.6^*^	<.001	.04	.10
	HII	+24.7 ± 11.6^*^, ^#^	+9.4 ± 9.3^*^			
Brachial SBP	MOD	+4.1 ± 14.4	+2.8 ± 10.5	.47	.13	.21
	HII	+0 ± 8.5	−4.7 ± 8.7^#^			
Brachial DBP	MOD	−0.1 ± 5.1	−1.4 ± 4.5	.07	.95	.19
	HII	+2.7 ± 6.4	−3.9 ± 8.6			
Aortic SBP	MOD	+2.1 ± 10.9	−0.7 ± 5.6	.07	.33	.38
	HII	+1.5 ± 6.9	−5.1 ± 8.1^*^			
Aortic DBP	MOD	+1.7 ± 5.8	−1.1 ± 5.8	.03	.52	.14
	HII	+6.4 ± 6.6^*^, ^#^	−2.6 ± 9.9			

Note

Data are presented as change in heart rate (bpm) and blood pressure (BP; mmHg) from baseline. Means ± *SD*. HII, high‐intensity interval exercise; MOD, continuous moderate intensity exercise.

^*^ *p* < .05 compared with baseline.

^#^ *p* < .05 compared with MOD.

### 3.3 Circulating microRNAs

**Figure 1:**
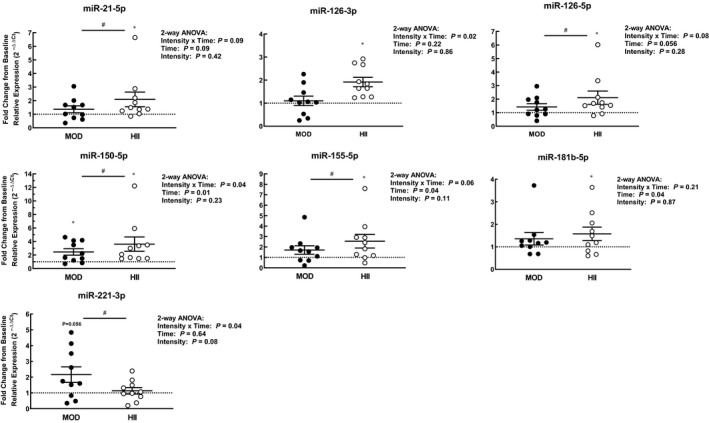
Fold‐changes relative to baseline (set at one) of circulating microRNAs (ci‐miRs) immediately after moderate continuous (MOD) or high intensity interval (HII) exercise. Means ± *SEM*, *n* = 10. ⁎*p* < .05 versus baseline levels, #*p* < .05 between intensities

No ci‐miRs or measures of arterial stiffness differed at baseline on the two testing sessions. There were significant interaction (time x intensity) effects on ci‐miRs‐ 126‐3p, 150‐5p, and 221‐3p, while there were significant time effects on ci‐miRs‐ 150‐5p, 155‐5p, and 181b‐5p (Figure 1). Additionally, there was a borderline significant time effect (*p* = .056) on ci‐miR‐126‐5p. Contrasts revealed significant increases immediately following HII exercise for all ci‐miRs studied except for ci‐miR‐221‐3p. Specifically, ci‐miR‐21‐5p increased by 109% (*p* = .005), ci‐miR‐126‐3p increased by 92% (*p* = .01), ci‐miR‐126‐5p increased by 111% (*p* = .003), ci‐miR‐150‐5p increased by 261% (*p* < .001), ci‐miR‐155‐5p increased by 155% (*p* = .001), and ci‐miR‐181b‐5p increased by 58% (*p* = .01) after HII exercise. In comparison, only ci‐miR‐150‐5p increased significantly after MOD exercise by 145% (*p* = .03), while ci‐miR‐221‐3p showed a borderline significant increase of 116% (*p* = .056). Post‐exercise expression levels of ci‐miRs‐ 21‐5p, 126‐5p, 150‐5p, and 155‐5p were significantly greater after the HII exercise bout compared to after the MOD exercise bout, while ci‐miR‐221‐3p was greater after the MOD bout versus after the HII bout (all *p* < .05). One subject showed large increases in several ci‐miRs in response to HII exercise compared to the rest of the subjects. All reported differences remained significant when this subject was excluded from analyses, except changes in ci‐miR‐181b‐5p following HII became borderline significant (*p* = .055).

### 3.4 Pulse wave velocity

**Figure 2:**
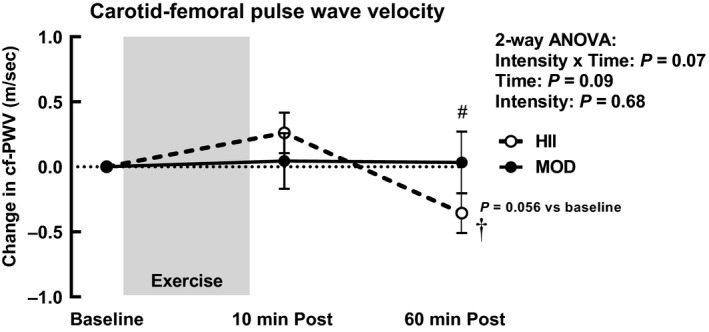
Change in carotid to femoral pulse wave velocity (cf‐PWV) 10 min after and 60 min after moderate continuous (MOD) or high intensity interval (HII) exercise relative to baseline. Means ± *SEM*, *n* = 9. †*p* < .05 versus 10 min post‐exercise, #*p* < .05 between intensities

Data for cf‐PWV, carotid β‐stiffness, and arterial compliance were not reliably measured during one subjects’ visit and are, therefore, presented from nine subjects. Two‐way ANOVA revealed borderline significant interaction (*p* = .07) and time effects on cf‐PWV (*p* = .09) (Figure 2). At 60 min post‐exercise, contrasts revealed a decrease in cf‐PWV after HII exercise compared to baseline that approached statistical significance (*p* = .056), and was significantly different compared to the value 60 min after MOD exercise (*p* = .04). The decrease in cf‐PWV 60 min after HII exercise was also significant in comparison to 10 min after exercise (*p* = .003). There were no significant effects of MOD exercise on cf‐PWV.

### 3.5 Carotid arterial compliance and β‐stiffness

**Figure 3:**
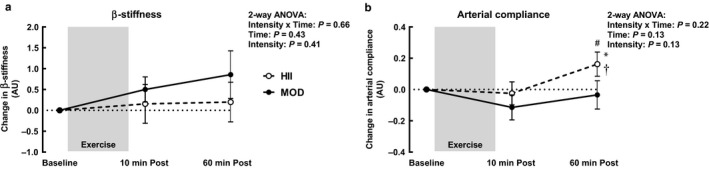
Carotid arterial β‐stiffness (a) and compliance (b) 10 min after and 60 min after moderate continuous (MOD) or high intensity interval (HII) exercise relative to baseline. Means ± *SEM*, *n* = 9. ⁎*p* < .05 versus baseline, †*p* < .05 versus 10 min post‐exercise, # *p* < .05 between intensities

Two‐way ANOVAs revealed no significant time, intensity, or interaction effects on carotid β‐stiffness (Figure 3a) or arterial compliance (Figure 3b). However, pre‐planned post hoc tests revealed a significant increase in arterial compliance 60 min after HII exercise compared to baseline (*p* = .049) and 10 min after exercise (*p* = .03). At 60 min post‐exercise, carotid arterial compliance was also higher after HII compared to MOD (*p* = .02). There were no significant effects of MOD exercise on carotid arterial compliance.

### 3.6 Augmentation index

**Figure 4:**
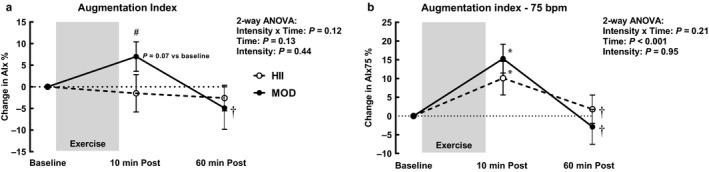
Augmentation index (AIx) (a) and AIx normalized to heart rate of 75 beats per minute (AIx75) (b) 10 min after and 60 min after moderate continuous (MOD) or high intensity interval (HII) exercise relative to baseline. Means ± *SEM*, *n* = 10. ⁎ *p* < .05 versus baseline, † *p* < .05 versus 10 min post‐exercise, # *p* < .05 between intensities

Despite no significant interaction, time, or intensity effects on AIx (Figure 4a), it trended toward increasing 10 min after MOD exercise (*p* = .07) and was also greater than at the same time point after HII exercise (*p* = .03). Sixty minutes after MOD exercise, AIx had decreased back towards baseline values (*p* = .004) and was no longer different to HII exercise. For AIx75 (Figure 4b), there was a significant time effect (*p* < .001), with a significant increase 10 min after both MOD (*p* < .001) and HII exercise (*p* = .02). One hour after exercise, AIx75 was significantly decreased compared to 10 min post‐exercise for both the MOD (*p* < .001) and HII (*p* = .04) bouts.

### 3.7 Correlations

Positive correlations were observed between peak exercise HR and changes in ci‐miRs −21‐5p (r = 0.60, *p* = .007), 126‐5p (r = 0.65, *p* = .003), and 150‐5p (r = 0.60, *p* = .006). Similar associations with average exercise HR were seen for the same ci‐miRs, 21‐5p (r = 0.63, *p* = .004), 126‐5p (r = 0.63, *p* = .004), and 150‐5p (r = 0.64, *p* = .003). Correlations between changes in ci‐miRs and measures of arterial stiffness are shown in Table 3. Changes in ci‐miRs‐ 21‐5p and 221‐3p correlated with the changes in cf‐PWV 10 and 60 min after exercise, respectively. There were also significant negative correlations between the changes in ci‐miRs‐ 21‐5p, 126‐3p, and 126‐5p with changes in AIx75 and/or AIx 10 min after exercise.

**Table 3:** Correlations between circulating microRNA and measures of arterial stiffness

	cf‐PWV		AIx		AIx75		β‐stiffness		Arterial Compliance	
	10 min	60 min	10 min	60 min	10 min	60 min	10 min	60 min	10 min	60 min
miR−21	r = .54	r = .09	r=−.60	r=−.19	r=−.53	r=−.24	r=−.23	r = .01	r=−.16	r = .11
	*p* = .02	*p* = .72	*p* = .006	*p* = .42	*p* = .02	*p* = .30	*p* = .34	*p* = .96	*p* = .50	*p* = .65
miR−126−3p	r = .29	r=−.02	r=−.50	r=−.15	r=−.48	r=−.02	r=−.42	r=−.39	r = .24	r = .10
	*p* = .23	*p* = .95	*p* = .02	*p* = .54	*p* = .03	*p* = .93	*p* = .08	*p* = .10	*p* = .33	*p* = .67
miR−126−5p	r = .44	r = .003	r=−.52	r=−.13	r=−.44	r=−.19	r=−.18	r = .07	r=−.22	r = .06
	*p* = .057	*p* = .99	*p* = .02	*p* = .58	*p* = .055	*p* = .42	*p* = .47	*p* = .77	*p* = .36	*p* = .82
miR−150	r = .24	r=−.16	r=−.09	r = .04	r=−.04	r=−.01	r=−.06	r=−.04	r=−.05	r = .25
	*p* = .33	*p* = .52	*p* = .71	*p* = .87	*p* = .87	*p* = .96	*p* = .80	*p* = .86	*p* = .84	*p* = .30
miR−155	r = .35	r=−.08	r=−.33	r=−.04	r=−.22	r = .001	r=−.37	r=−.13	r=−.10	r = .14
	*p* = .15	*p* = .76	*p* = .15	*p* = .86	*p* = .34	*p* = .99	*p* = .12	*p* = .59	*p* = .70	*p* = .56
miR−181b	r = .16	r=−.13	r=−.10	r=−.03	r=−.03	r=−.008	r=−.38	r=−.11	r = .19	r = .39
	*p* = .52	*p* = .58	*p* = .68	*p* = .91	*p* = .89	*p* = .97	*p* = .11	*p* = .65	*p* = .44	*p* = .10
miR−221	r = −.02	r = .48	r=−.03	r=−.34	r=−.27	r=−.33	r = .31	r = .19	r=−.26	r=−.23
	*p* = .92	*p* = .04	*p* = .89	*p* = .15	*p* = .25	*p* = .16	*p* = .20	*p* = .43	*p* = .28	*p* = .34

Correlations between changes in circulating microRNAs immediately after and measures of arterial stiffness 10 and 60 min (min.) after both moderate and high intensity interval exercise (*n* = 19–20). cf‐PWV, carotid to femoral pulse wave velocity; AIx, augmentation index; AIx75, augmentation index normalized to 75 beats per minute.

## 4 DISCUSSION

The novel findings of the present study are that (a) a set of vascular‐related ci‐miRs displayed distinct exercise intensity‐dependent increases, (b) 60 min after HII exercise, cf‐PWV was numerically decreased (*p* = .056) and carotid arterial compliance was increased compared to baseline, and both were different compared to the same time point after MOD exercise, (c) MOD exercise had no effect on these measures of central and large artery stiffness, (d) AIx75 was increased 10 min after both HII and MOD exercise and returned to baseline by 60 min of recovery, and (e) there were select significant correlations between changes in ci‐miRs with cf‐PWV and AIx/AIx75.

### 4.1 Arterial stiffness and wave reflection

In agreement with previous studies, we found no change in cf‐PWV 10 min after exercise regardless of intensity (Peres et al., 2018; Siasos et al., 2016; Tordi, Mourot, Colin, & Regnard, 2010). Siasos et al. (2016) likewise found no changes in cf‐PWV 10 min after either a 30 min HII cycling bout including repeated 30 s maximal sprints, or a 30 min continuous bout at 50% VO_2max_. Other studies using matched HII and MOD cycling protocols eliciting average HRs lower than those in our current study (~132 bpm (Peres et al., 2018) and ~ 143 bpm (Tordi et al., 2010)) also found no immediate changes in central or upper limb PWV (Peres et al., 2018; Tordi et al., 2010). However, we did observe a significant decrease in cf‐PWV 60 min compared to 10 min after HII exercise, which was also numerically different to baseline values and approached statistical significance. At the 60 min post‐exercise time point, cf‐PWV was also significantly lower following HII exercise as compared to MOD exercise. Carotid arterial compliance exhibited a corresponding increase 60 min into recovery following HII exercise, at which point it was also greater in comparison to MOD exercise. Indeed, there were no significant changes in either cf‐PWV or carotid arterial compliance in response to MOD exercise. In combination, our data suggest that HII, but not MOD, exercise exerts beneficial acute effects on central and large arterial stiffness that are apparent by one hour of recovery.

AIx may change independently of arterial stiffness and gives an indication of afterload imposed on the left ventricle due to wave reflection (Kim & Braam, 2013). AIx generally decreases immediately after exercise, however this is likely mediated by increased HR (Mutter et al., 2017; Pierce et al., 2018). When normalized to a HR of 75 bpm, AIx75 typically increases immediately after exercise (Mutter et al., 2017; Pierce et al., 2018). In the present study AIx75 was similarly increased 10 min after both MOD and HII exercise. Other protocols comparable to ours, though employing running on a treadmill, resulted in increased AIx75 five minutes after both HII and MOD exercise that was greater following the HII bout (Hanssen et al., 2015). Following both bouts, AIx75 had returned to baseline by 50 min of recovery (Hanssen et al., 2015). Likewise in the present study, AIx75 decreased back to baseline by 60 min of recovery in comparison to 10 min after both exercise intensities. Taken together, the data suggest that central arterial stiffness may have a limited effect on changes in wave reflection, and other factors, namely sympathetically driven peripheral vasoconstriction, may contribute to changes in wave reflection in our study (Atkinson, Lewis, et al., 2015; Protogerou & Safar, 2007).

The specific mechanisms mediating changes in arterial stiffness with acute exercise are not completely understood, and others have shown that changes are independent of alterations in HR and blood viscosity (Kingwell, Berry, Cameron, Jennings, & Dart, 1997; Naka et al., 2003). There were significant changes in aortic BP following HII exercise that did not occur following MOD exercise in our study, and these differences may have contributed to the different responses in central arterial stiffness. We have previously reported no intensity‐dependent differences in either brachial or aortic mean arterial pressures following exercise in these same subjects (Sapp et al., 2019). Furthermore, exercise‐induced changes in arterial stiffness have been reported to occur independently of changes in mean arterial pressure, so it is likely that other mechanisms primarily contributed to the changes in arterial stiffness in our study (Heffernan, Collier, Kelly, Jae, & Fernhall, 2007; Kingwell et al., 1997; Tordi et al., 2010).

Shear stress is another hemodynamic factor that displays a unique response to HII and MOD exercise, and may therefore account for divergent changes in arterial stiffness. High intensity interval exercise elicits greater shear rate along the vessel wall as compared with a matched bout of moderate continuous exercise (Atkinson, Carter, et al., 2015; Johnson & Wallace, 2012; McManus, Sletten, & Green, 2019). The pattern and magnitude of shear stress is important in regulating endothelial cell expression of miRs (Donaldson et al., 2018; Lee & Chiu, 2019) and it was recently shown that increased shear stress elicits the release of miR from cultured endothelial cells that are also increased in circulation in response to acute exercise ( Schmitz et al., 2019). Other factors that could contribute to changes in arterial stiffness include acute elevations in inflammation and ROS, stimuli known to increase with exercise in an intensity‐dependent manner (Allen, Sun, & Woods, 2015; Johnson et al., 2012; McClean et al., 2015; Vlachopoulos et al., 2005; Vucinovic et al., 2015). Both of these factors are also known regulators of miR expression and release by vascular cells (Alexy et al., 2014; Donaldson et al., 2018; Lee & Chiu, 2019), suggesting ci‐miRs may contribute to exercise‐induced changes in arterial stiffness.

### 4.2 Circulating micrornas

The sensitivity of ci‐miRs to different exercise parameters has recently been investigated by a number of studies (reviewed in Sapp & Hagberg, 2019). We have added to the literature by identifying exercise intensity‐dependent increases in a set of vascular‐related ci‐miRs. With regard to the underlying mechanistic purpose for changes in ci‐miR concentrations with exercise, two major hypotheses have been proposed (Sapp & Hagberg, 2019). First, in response to exercise stimuli, cells may secrete miRs as a means of offloading them in order to allow increased intracellular translation of their mRNA targets. Secondly, cells may be releasing ci‐miRs as a method of intercellular paracrine or endocrine communication in order to induce responses in target cells. There is substantial evidence supporting the transport of biologically active ci‐miRs between endothelial cells and from endothelial to vascular smooth muscle cells (Bär, Thum, & Gonzalo‐Calvo, 2019; Hergenreider et al., 2012; Jansen et al., 2013, 2015, 2017; Lemcke & David, 2018; Njock & Fish, 2017; Zhou et al., 2013). Either of these mechanisms could explain how ci‐miRs mediate adaptations to exercise training, due to either repeated offloading or transport with acute exercise bouts. Given experimental support for intercellular transport of ci‐miR, we hypothesize that acute alterations in ci‐miR act primarily in a paracrine/endocrine manner. An additional potential mechanism of increased ci‐miR is that of passive release due to exercise‐induced cell damage, although this does not appear to be the case at least in regard to the endothelium ( Sapp et al., 2019).

Notably, Ramos et al. recently identified ci‐miRs with either threshold or dose‐response kinetics to exercise by having men run at variable speeds or durations, while holding the other variable constant ( Ramos et al., 2018). The only ci‐miR also tested in our study, ci‐miR‐21‐5p, failed to increase and, therefore, did not show an intensity‐dependent response in their study ( Ramos et al., 2018). Conversely, we observed no change in ci‐miR‐21‐5p in response to a MOD exercise bout, but a ~ two‐fold increase in response to a HII exercise bout. Similar HII‐specific increases were seen for ci‐miRs‐ 126‐3p, 126‐5p, 155‐5p, and 181b‐5p. Only ci‐miR‐150‐5p was significantly increased in response to both exercise bouts, though to a greater degree after HII exercise. Lastly, ci‐miR‐221‐3p showed a borderline significant increase specifically in response to MOD exercise, and post‐exercise levels were greater compared to after HII exercise. A major strength of our study compared to previous ones is that our exercise bouts were matched for overall time and volume.

Ci‐miR‐126‐3p is the predominant endothelial miR and is among the best characterized ci‐miR in response to exercise (Fish et al., 2008; Harris et al., 2008; Sapp et al., 2017,; Wang et al., 2008). As previously reported, we observed an increase in ci‐miR‐126‐3p following HII exercise with no change following MOD exercise ( Sapp et al., 2019). Others have reported conflicting effects of exercise intensity (Kilian et al., 2016; Wahl et al., 2016). The contrasting results of our study compared to previous studies may stem from our substantially shorter MOD bout, the fact that our subjects were all recreationally active men, and/or differences in sampling types and timing used between studies (Kilian et al., 2016; Wahl et al., 2016).

From a mechanistic perspective, miR‐126‐3p is considered a master regulator of endothelial cell function, targeting the VEGF and SDF‐1 pathways to influence angiogenesis and NO bioavailability, as well as influencing vascular inflammation and ROS through various targets ( Chistiakov, Orekhov, & Bobryshev, 2016). It is released into circulation by endothelial cells and is internalized by both endothelial and vascular smooth muscle cells (Jansen et al., 2013, 2015, 2017; Njock & Fish, 2017; Zhou et al., 2013). Thus, miR‐126‐3p could regulate arterial stiffness via its effects on vascular inflammation, NO, and ROS, potentially via direct downregulation of VCAM‐1 and upregulation of SIRT1 expression (Chistiakov et al., 2016; Harris et al., 2008; Zhou et al., 2013). We hypothesize that ci‐miR may be elevated immediately in response to exercise and may be taken up by the vasculature where they can affect post‐exercise arterial stiffness. Therefore, we analyzed correlations between immediate changes in ci‐miR and changes in measures of arterial stiffness 10 and 60 min after exercise. In addition to the previously reported negative correlation between exercise‐induced change in ci‐miR‐126‐3p and the FMD response 10 min after MOD exercise ( Sapp et al., 2019), here we observed a negative correlation with the change in AIx and AIx75 10 min after exercise (including both intensities). The complimentary miR‐126‐5p strand is also highly expressed within endothelial cells and is biologically active (Fish et al., 2008; Kumar, Williams, Sur, Wang, & Jo, 2019; Schober et al., 2014). Ci‐miR‐126‐5p increased in response to HII exercise similarly to the miR‐126‐3p strand and was also inversely associated with the change in AIx/AIx75 10 min after exercise. Thus, both strands of ci‐miR‐126 may play roles in the regulation of wave reflection after exercise.

Additionally, ci‐miRs‐ 150‐5p, 155‐5p, and 181b‐5p increased following HII exercise, while only ci‐miR‐150‐5p also increased in response to MOD exercise. MiRs‐ 150, 155, and 181b are anti‐inflammatory via indirect regulation of NF‐kB (Cheng, Njock, Khyzha, Dang, & Fish, 2014; Luo et al., 2018). MiR‐155 may also have pro‐inflammatory effects, and acts to regulate smooth muscle cell function by directly targeting eNOS and angiotensin II type I receptor expression (Sun et al., 2012; Yang et al., 2014; Zhang, Zhao, Yu, Lu, & Zheng, 2015). The greater and/or specific increases in these ci‐miRs after HII could both reflect and perpetuate a greater anti‐inflammatory response to the HII exercise bout as compared to the MOD bout, although we found no significant correlations between changes in these ci‐miRs and measures of arterial stiffness.

Ci‐miR‐221‐3p expression was significantly greater after MOD exercise compared to after the HII bout. Within endothelial and smooth muscle cells, miR‐221 is anti‐angiogenic and anti‐inflammatory (Celic, Metzinger‐Le Meuth, Six, Massey, & Metzinger, 2016; Chistiakov, Sobenin, Orekhov, & Bobryshev, 2015). It can also increase ROS production by directly targeting PGC‐1α and adiponectin receptor I (Chen et al., 2015; Xue et al., 2015). Changes in ci‐miR‐221‐3p correlated positively with the change in cf‐PWV 60 min after exercise, while only changes in ci‐miR‐21‐5p were positively associated with the change in cf‐PWV 10 min after exercise. Interestingly, ci‐miR‐21‐5p, which increased specifically in response to HII exercise and was greater compared to levels after MOD exercise, may have similar effects on angiogenesis and ROS production as miR‐221 ( Chamorro‐Jorganes, Araldi, & Suarez, 2013). Thus, the different levels of these ci‐miRs after MOD compared to HII exercise may hypothetically contribute to the exercise‐intensity dependent differences in arterial stiffness.

There are some limitations of our study that should be mentioned. Ci‐miRNA were only analyzed immediately after exercise, so it is possible that we missed further elevations in circulation. However, previous studies suggest that ci‐miRNA expression peaks soon after exercise and returns to baseline by one hour of recovery (reviewed in 55 and 56). In addition, arterial stiffness measures were obtained 10 min after exercise, so more immediate effects of exercise may have been missed ( Mutter et al., 2017). There are reported effects of sex, CVD risk factors, and training status on circulating microRNAs and arterial stiffness, so only young, healthy men were included as participants in this initial study, limiting the generalizability of our results to other populations (Ameling et al., 2015; Bye et al., 2013; Cui et al., 2018; Jones Buie, Goodwin, Cook, Halushka, & Fan, 2016).

In conclusion, we have identified unique changes and associations in concentrations of vascular‐related ci‐miRs and measures of arterial stiffness following a HII and a MOD exercise bout. Our results suggest a potential intensity‐dependent threshold for miR release from cardiovascular cells that may play a role in acute beneficial changes in arterial stiffness specifically after HII. These intensity‐dependent effects could also have implications for the mechanisms underlying long‐term training adaptations in arterial health, although the observed effects of acute exercise cannot be extrapolated to effects of exercise training. Determining the roles of ci‐miRs in exercise‐induced changes in arterial stiffness may inform the development of future therapeutic strategies.

## CONFLICT OF INTERESTS

The authors report no potential conflicts of interest.

## AUTHOR CONTRIBUTIONS

RMS, SMR, and JMH conceived and designed experiments; RMS, CAC, LEE, WSE, EMZ, and SMR collected data; RMS, CAC, LEE, WSE, EMZ, SJP, SMR, and JMH analyzed data and interpreted results; RMS drafted the manuscript and prepared figures; RMS, CAC, LEE, WSE, EMZ, SJP, SMR, and JMH edited, revised, and approved the final version of the manuscript. All authors agree to be accountable for all aspects of the work in ensuring that questions related to the accuracy or integrity of any part of the work are appropriately investigated and resolved. All persons designated as authors qualify for authorship, and all those who qualify for authorship are listed.
